# Autophagy Targeting and Hematological Mobilization in FLT3-ITD Acute Myeloid Leukemia Decrease Repopulating Capacity and Relapse by Inducing Apoptosis of Committed Leukemic Cells

**DOI:** 10.3390/cancers14020453

**Published:** 2022-01-17

**Authors:** Marine Dupont, Mathilde Huart, Claire Lauvinerie, Audrey Bidet, Amélie Valérie Guitart, Arnaud Villacreces, Isabelle Vigon, Vanessa Desplat, Ali El Habhab, Arnaud Pigneux, Zoran Ivanovic, Philippe Brunet De la Grange, Pierre-Yves Dumas, Jean-Max Pasquet

**Affiliations:** 1 Cellules Souches Hématopoïétiques Normales et Leucémiques, INSERM U1312 BRIC, Université de Bordeaux, Bat TP 4e étage, 146 rue Léo Saignat, 33076 Bordeaux, France; marine148@hotmail.fr (M.D.); mathilde.huart@u-bordeaux.fr (M.H.); claire.lauvinerie@u-bordeaux.fr (C.L.); audrey.bidet@chu-bordeaux.fr (A.B.); amelie.guitart@u-bordeaux.fr (A.V.G.); arnaud.villacreces@u-bordeaux.fr (A.V.); isabelle.vigon@u-bordeaux.fr (I.V.); vanessa.desplat@u-bordeaux.fr (V.D.); ali.el-habhab@u-bordeaux.fr (A.E.H.); arnaud.pigneux@chu-bordeaux.fr (A.P.); Zoran.Ivanovic@efs.sante.fr (Z.I.); Philippe.Brunet-De-La-Grange@efs.sante.fr (P.B.D.l.G.); pierre-yves.dumas@u-bordeaux.fr (P.-Y.D.); 2 Service d’Hématologie Biologique, CHU Bordeaux, 33000 Bordeaux, France; 3 Service d’Hématologie Clinique et Thérapie Cellulaire, CHU Bordeaux, 33000 Bordeaux, France; 4 Etablissement Français du Sang Nouvelle Aquitaine, 33035 Bordeaux, France

**Keywords:** acute myeloid leukemia, FLT3-ITD, tyrosine kinase inhibitors, persistence, leukemic initiating cells, autophagy

## Abstract

**Simple Summary:**

One of the most frequent molecular anomalies in acute myeloid leukemia (AML) is the mutation of the fms-like receptor tyrosine kinase 3 through internal tandem duplications, giving rise to a constitutive proliferative signaling. Even though clinical trials have shown that targeting this mutated kinase is of interest and well tolerated, there is still a high frequency of relapse. The emergence of AML cells upon treatment is linked to their maintenance through resistance and persistence mechanisms. Because FLT3-ITD AML cells require autophagy, we explored the consequence of autophagy inhibition by blocking the PI3-kinase class III, Vps34, when AML cells were committed. Results in vitro, ex vivo and in vivo suggest that remission with low minimal residual disease in FLT3-ITD AML offers a promising therapeutic window to target persistent leukemic cells.

**Abstract:**

Targeting FLT3-ITD in AML using TKI against FLT3 cannot prevent relapse even in the presence of complete remission, suggesting the resistance and/or the persistence of leukemic-initiating cells in the hematopoietic niche. By mimicking the hematopoietic niche condition with cultures at low oxygen concentrations, we demonstrate in vitro that FLT3-ITD AML cells decrease their repopulating capacity when Vps34 is inhibited. Ex vivo, AML FLT3-ITD blasts treated with Vps34 inhibitors recovered proliferation more slowly due to an increase an apoptosis. In vivo, mice engrafted with FLT3-ITD AML MV4-11 cells have the invasion of the bone marrow and blood in 2 weeks. After 4 weeks of FLT3 TKI treatment with gilteritinib, the leukemic burden had strongly decreased and deep remission was observed. When treatment was discontinued, mice relapsed rapidly. In contrast, Vps34 inhibition strongly decreased the relapse rate, and even more so in association with mobilization by G-CSF and AMD3100. These results demonstrate that remission offers the therapeutic window for a regimen using Vps34 inhibition combined with mobilization to target persistent leukemic stem cells and thus decrease the relapse rate.

## 1. Introduction

Acute myeloid leukemia (AML) is a malignant hematopoietic disorder in which blockage of differentiation and high proliferation lead to the accumulation of nonfunctional blood cells. The Fms-like tyrosine kinase 3 (FLT3) gene encodes a class III receptor tyrosine-kinase (RTK) that is expressed in hematopoietic stem progenitor cells and that signals through PI3K/AKT and MAPK pathways upon ligand binding [1]. Internal tandem duplication (ITD) in FLT3 is the most frequent mutation found in acute myeloid leukemia (AML) with normal karyotype [2,3]. Although the FLT3-ITD mutation is a late event in leukemogenesis, it is an important target for the disease [4,5]. FLT3-ITD confers a poor prognosis in adult AML patients, and its frequent occurrence at relapse suggests that FLT3-ITD AML leukemic initiating cells (LIC) are key targets for long-lasting remission [6]. Recently, new FLT3 tyrosine kinase inhibitors have been developed. These new TKIs are well tolerated, allow the treatment of older AML patients and induce complete remission with low minimal residual disease. However, the TKI against FLT3 used in AML treatment cannot prevent relapse even in the presence of complete remission, thus suggesting the resistance and/or the persistence of a LIC compartment via different mechanisms [7,8].

Because so many mechanisms participate in the maintenance of the LIC, the challenge is to inhibit all of them while sparing normal hematopoiesis. FLT3-ITD is no longer expressed in hypoxia, such as inside the bone marrow [9,10,11]. However, it has been detected in the immature CD34^+^/CD38-/CD123^+^ fraction in AML [12,13]. The size of this immature compartment is reported prognostic of an adverse outcome for AML patients [14]. Indeed, several clinical trials concluded that second-line treatments have to be as strong as possible to reach and sustain complete remission [15]. Furthermore, hypoxia limits the response to chemotherapy, hence the recent development of specific hypoxia-activated FLT3 inhibitors [16]. In addition, AML stem cells were detected in different phenotypic compartments, suggesting a workflow hardly targetable, and autophagy was suspected to play roles in resistance to FLT3-TKI [17,18]. Indeed, several studies have reported that the reservoir of stem cells residing within the hematopoietic niche may not be visible without forcing their exit and engagement in hematopoiesis [19,20]. In addition, in AML it has been reported that pushing out LIC from the protective niche prevents their persistence and resistance [21,22,23]. We hypothesized, knowing that normal progenitors are insensitive to Vps34 inhibitors, that an inhibition of Vps34 associated with the mobilization of normal and leukemic stem cells would unmask the latter and eliminate them by blocking autophagy [24]. In summary, it seems that too many mechanisms specific to the hematopoietic niche are involved in pathways decreasing response to treatment to expect that targeting only few of them will be efficient [25,26].

Because hematopoietic stem cells (HSC) produce a continuous stream of differentiated blood cells, it would be more efficient to inhibit the mechanism at the initial stage when small amounts of repopulating cells leave the hematopoietic niche. The idea is to take advantage of the minimal disease upon remission to force the CSH to engage so as, at the same time, to attract the residual LIC, which are sensitive to the inhibition of autophagy. The level of the minimal residual disease differs from one patient to another. The question is how to target only LIC efficiently and to eradicate them. We used a model with low O_2_ concentration growing conditions to mimic the hypoxic state inside the hematopoietic niche, where cells are low cycling or quiescent, and replaced them with cells that have a higher oxygen concentration. Replacing CD34^+^ progenitors of chronic myeloid leukemia in growing conditions is deleterious if autophagy is blocked by the inhibition of the initiating step involving the PI3-kinase class III, Vps34 [24], while sparing normal CD34^+^ progenitors. Autophagy is induced in FLT3-ITD AML cells by the deregulated FLT3 through endoplasmic reticulum stress and the ATF4 transcription factor [27]. This demonstrates that a constitutively activated TKI, which triggers mTor activation, can also induce autophagy via several pathways, as already reported in colorectal cancer [28]. Autophagy is required for the proliferation of FLT3-ITD cells, while non-mutated cells are not sensitive. In FLT3-ITD cells, the inhibition of proteasome by bortezomib induced an autophagy-dependent degradation of FLT3, leading to the apoptosis of leukemic cells [29]. In addition, autophagy has been implicated in the self-renewal and quiescence of HSC and progenitor differentiation [30,31,32]. All of these suggest the potential benefit of targeting autophagy in FLT3-ITD cells.

In this study, we aimed at investigating whether autophagy is dispensable when FLT3-ITD cells leave the hematopoietic niche to commit and induce relapse. To answer this, we used a previously described strategy, in which cells are placed at low concentration of oxygen mimicking the intra-bone marrow conditions, allowing low cycling and then replaced at an atmospheric oxygen concentration, which led to proliferation and repopulation. Pharmacological inhibition of autophagy was performed in vitro, ex vivo and in vivo using the PI3-kinase class III Vps34 inhibitors, PIK-III and SAR-405, in combination or not, to mobilization using AMD3100 and G-CSF [33,34,35,36].

## 2. Materials and Methods

### 2.1. Reagents

The αMEM medium, fetal calf serum (FCS) and phosphate buffered saline (PBS) were from Invitrogen. The trypan blue and the anti-LC3 antibody were from Sigma (St Quentin Fallavier, France). The annexin-V-APC was from Biolegend (France). The PIK-III was purchased from Selleck Chemicals (Houston, TX, USA). Cell apoptosis was assessed using an APC-conjugated annexin V labeling detection kit coupled to flow cytometry and BDFACSDIVA^TM^ software Becton Dickinson, Le Pont de Claix, France. The murine G-CSF was from Peprotech (Neuilly, France) and AMD3100 from SantaCruz (Heidelberg, Germany). Gilteritinib and SAR-405 were purchased from MedChemTronica (Sollentuna, Sweden).

### 2.2. Cell Lines

All cell lines (MV4-11, MOLM-14 and Oci-AML3) were from the *American Type Culture Collection* (ATCC) and were cultured in an αMEM medium supplemented with 10% fetal calf serum (FCS), 2 mM L-glutamine, 50 U/mL penicillin and 50 µg/mL streptomycin. The low O_2_ concentration was induced by incubating cells in a specific O_2_ chamber (Parish, Towns in Oswego Count, NY, USA). Aliquots were taken at 24 h intervals to assess cell viability by trypan blue exclusion.

### 2.3. AML Patient Biological Samples

AML samples were obtained from the biological resource center at the University Hospital of Bordeaux for patients who gave written informed consent for the use of biological samples for research, in accordance with the Declaration of Helsinki, allowing the collection of clinical and biological data in an anonymized database registered at the Commission Nationale de l’Informatique et des Libertés (N°915285 authorization) and CPP (N°2015-08-11D authorization). Bone marrow mononuclear cells were purified and frozen until thawed for experiments. They were co-cultured with MS5 stromal cells in stem span medium (Stem Cell Technologies, Saint Egreve, France), unless otherwise stated.

### 2.4. Culture at Low or Atmospheric Oxygen Concentration

**Figure 1:**
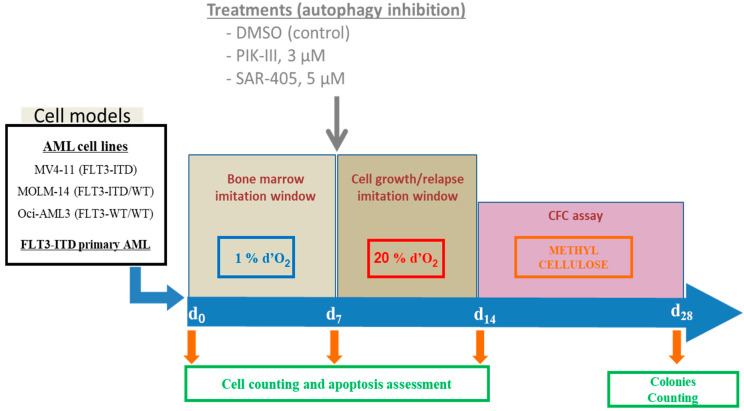
Schematic presentation of experimental procedure. AML cell lines growing were placed at day 0 at 1% O_2_ for 7 days. At day 7, cells were counted and apoptosis was quantified by flow cytometry using annexin V labeling. Then, cells were treated or not and placed at 20% O_2_ for the next 7 days. Then, cell count and apoptosis were measured before seeding 10^3^ cells in a methylcellulose dish. After 14 days, colonies were quantified by microscopy counting in triplicate.

As reported by Ianicieillo et al., cells were placed at day zero (d_0_) at low oxygen concentration (1% O_2_ for 7 days in an Xvivo System culture hood (BioSpherix, Parish, NY, USA). After 7 days (d_7_), cells were replaced at atmospheric oxygen concentration for 7 days until day 14. At days 0, 7 and 14, aliquots were analyzed for cell number and apoptosis as presented in the first part of Figure 1. After 14 days, 10^3^ viable cells were seeded in methylcellulose and colonies were counted 14 days later to quantify the CFC assessing immaturity and repopulating capacity.

### 2.5. Flow Cytometry

Cells (10^5^ cells) were incubated for 10 min in 500 µL of Hepes/NaCl buffer with 2 mM Ca^2+^, 2 µL of Annexin V- APC and 0.25 µg of propidium iodine (PI) before flow cytometry on a Facs Canto II. At least 10,000 events were acquired for statistical analysis. Detection of apoptosis by annexin V labeling was performed according to the manufacturer’s instructions (Biolegend, Paris, France).

### 2.6. Autophagy Inhibition by PIK-III and SAR-405

PIK-III and SAR-405 were dissolved in DMSO at 20 mM. A dose response experiment was performed to determine the concentration that inhibits autophagy with minimal apoptosis using AML cells expressing the mCherry-GFP-LC3 protein as a fluorescent reporter of autophagy (kindly provided by Dr Soengas, Madrid, Spain). AML cells were incubated with 0, 0.3, 1, 3, 5, 10 and 20 µM of inhibitors for 24, 48 and 72 h and cells were analyzed by flow cytometry after annexin V-APC labeling. During the autophagy process, LC3 is present in autophagolysosomes and is then degraded at low pH. We used the mCherry-GFP-LC3 construction, which has a dual fluorescence when autophagy is inhibited. PIK-III and SAR-405 increased GFP and mCherry fluorescence in a dose-dependent manner. Regarding the concentration inhibiting autophagy with no significant apoptosis, 3 and 5 µM were chosen for PIK-III and SAR-405, respectively.

### 2.7. Western Blotting

Protein lysates were prepared according to Weisberget al. [37,38]. Protein concentration was measured by the BCATM Protein Assay (Pierce, Rockford, IL, USA) and the lysates were stored at –80 °C. Equal amounts of protein were separated by electrophoresis on an SDS-PAGE 12.5 and transferred to a pvdf membrane, as described [39] (Biorad, Marnes-La-Coquette, France). After blocking, the membrane is incubated with primary antibodies and secondary antibodies. Protein–antibody complexes were detected by an enhanced chemiluminescence immunoblotting ECL (Biorad, Marnes-La-Coquette, France). The antibodies used are described in Supplementary Materials.

### 2.8. Animal Models for In Vivo Studies

NOD Cg-Prkdc^scid^ Il2rg^tm1Wjl^/SzJ immunodeficient mice (NSG) were bred at the University of Bordeaux’s A2 animal facility for experiments validated by the French ministry (N°00048.2 authorization). Animals were included in protocols at eight weeks old and were monitored weekly for body weight. For hematopoietic engraftment, female NSG mice were pretreated with intraperitoneal injections of busulfan (Fabre) 20 mg/kg/day for two days, followed by an intravenous injection of MV4-11 luciferase-transduced cells (5 × 10^5^ cells/100 μL) at day 0. Each week, the engraftment was analyzed by whole bioluminescent imaging (BLI), as reported [11]. At day 14, engraftment was analyzed by whole BLI before treating mice with the vehicle or gilteritinib (30 mg/kg/day body weight) by daily oral gavage. Upon 4 weeks treatment, mice were randomized and treated for 2 days as follows: 1-NT: intraperitoneal injection of vehicle, SAR (SAR-405 10 µM IP), MSAR (SAR-405 10 µM, G-CSF 125 µg/kg, AMD3100 5 mg/mL IP), PIK (PIK 25 mg/kg gavage) and MPIK (PIK 25 mg/kg gavage, G-CSF 125 µg/kg IP, AMD3100 5 mg/mL IP). Mice were monitored each day and weekly by bioluminescence imaging.

### 2.9. Statistical Analysis

All analyses were performed using GraphPad Prism software. A Wilcoxon test was used to calculate the differences between means. Differences were considered significant only when *p* ≤ 0.05 or 0.01 as indicated and shown by an asterisk * or **. ns means not significant.

## 3. Results

### 3.1. Vps34 Inhibition Using PIK-III and SAR-405 Block Autophagy in AML Cells

Two Vps34 inhibitors were used for flow cytometry in a dose- and time-dependent manner to detect autophagy inhibition using MV4-11, MOLM-14 and Oci-AML3 cell lines expressing the mCherry-GFP-LC3 protein as a fluorescent reporter of autophagy. Supplementary Figure S1A–C shows that both mCherry and GFP fluorescence increased in each AML cell line treated for 24 h by increasing doses of PIK-III or SAR-405. This inhibition was both confirmed by the detection of mCherry and GFP fluorescence by microscopy, as shown in Supplementary Figure S1D, and the autophagy markers p62, Beclin and LC3 by Western blotting in Supplementary Figure S1E.

To test the consequence of autophagy inhibition in AML cells leaving quiescence, we first incubated AML cells at 1% O_2_ for 7 days to place them in a similar condition as AML cells in the bone marrow at low O_2_ concentration (Figure 1). At day 7, cells were counted and apoptosis was quantified by flow cytometry using annexin V labeling. Cells were brought back to 20% O_2_ with or without Vps34 inhibitor (PIK-III 3 µM or SAR-405 5 µM). At day 14, cells were counted and apoptosis was again quantified.

### 3.2. Inhibition of Autophagy Reduces Expansion and Enhances Apoptosis of FLT3-ITD AML Cells Following Transfer to Growth-Permissive Cultures at Atmosphere O_2_ Concentration

**Figure 2:**
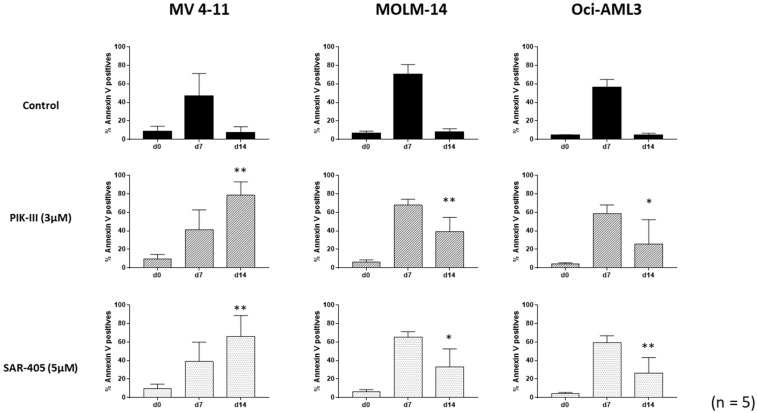
Inhibition of autophagy enhances apoptosis of FLT3-ITD AML cells. AML cell lines were placed at day 0 at 1% O_2_ for 7 days. At day 7, cells were counted and apoptosis was quantified. Then, cells were treated or not and placed at 20% O_2_ for the next 7 days. At indicated times (d_0_, d_7_ and d_14_), aliquots were analyzed by flow cytometry using annexin V-APC labeling. Results are from at least 5 experiments. Significance was quantitated using Wilcoxon test and is shown by an asterisk (* *p* < 0.05, ** *p* < 0.01).

Following the procedure shown in Figure 1, we quantified apoptosis in each condition in a time course experiment for the three AML cell lines (Figure 2). Apoptosis had increased in each cell line after 7 days at 1% O_2,_ as normally observed in such culture conditions. At 20% O_2_, each cell line grew and apoptosis returned to baseline (Figure 2, line control).

In contrast, inhibition of autophagy by both PIK-III and SAR-405 led to a large increase in apoptosis in FLT3-ITD MV4-11 cells at day 14. This increase was accompanied by a decrease in cell number, as shown in Supplementary Figure S2A, and an apoptotic signaling was detected through PARP and caspase 3 cleavage by Western blotting (Supplementary Figure S2B). Interestingly, the partial decrease in apoptosis for both MOLM-14 and Oci-AML3 cells at day 14 when treated with Vps34 inhibitors suggests the benefit of autophagy inhibition, independently of FLT3-ITD.

### 3.3. Inhibition of Autophagy Limits Repopulating Capacity of FLT3-ITD Cells in CFC Assay

**Figure 3:**
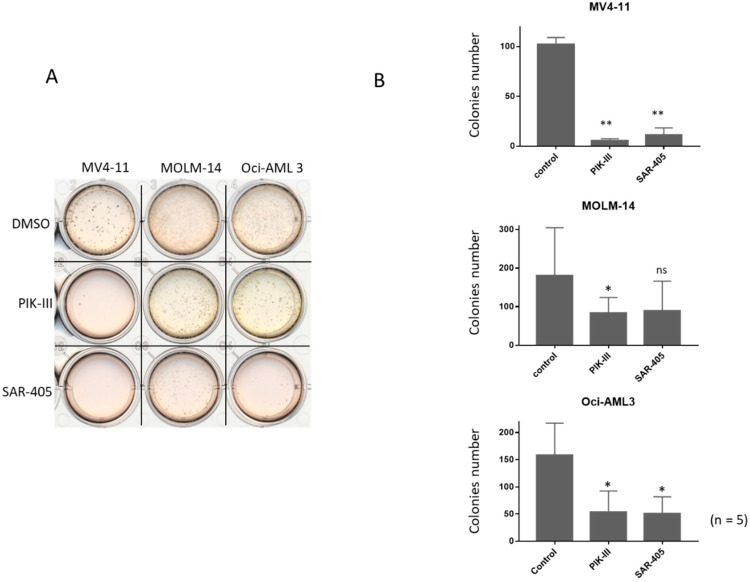
Inhibition of autophagy reduces repopulating capacity and colony number of FLT3-ITD AML cells. AML cells were incubated for 7 days at 1% O_2_ and then treated or not for 7 more days at 20% O_2_. After cell counting at d14, 10^3^ cells were seeded in methylcellulose and incubated at 37 °C at 20% O_2_. After 14 days in methyl cellulose, colony number was quantified. (**A**) A representative observation of colonies in methylcellulose. (**B**) Results from at 5 experiments are shown. Significance was quantitated using Wilcoxon test and is shown by an asterisk (ns: not significant, * *p* < 0.05, ** *p* < 0.01).

To evaluate the consequence of autophagy inhibition on the repopulating capacity of FLT3-ITD AML cells, we seeded at day 14 in methylcellulose 10^3^ viable cells from the untreated condition or incubated between day 7 and 14 with one addition of PIK-III or SAR-405. Figure 3A shows that in the untreated condition, repopulating capacity was great for each AML cell line after 7 days at 1% O_2_ followed by 7 days at 20% (Figure 3A, condition DMSO).

Five independent experiments showed that FLT3-ITD AML cells significantly lost their repopulating capacity by autophagy inhibition (Figure 3B). Strikingly, MOLM-14 and Oci-AML3 cells also had a decrease of their repopulating capacity when autophagy was inhibited (Figure 3B), although it was weaker than in MV4-11. Therefore, treatment with PIK-III or SAR-405 strongly inhibits the repopulating capacity of FLT3-ITD cells after 7 days at 1% O_2_. This is inhibition is lesser in MOLM-14 and Oci-AML3. Supplementary Figure S3A,B shows colonies after 14 days in methylcellulose.

### 3.4. Ex Vivo Autophagy Inhibition of Primary FLT3-ITD AML Cells Increases Apoptosis upon Transfer to Growth-Permissive Cultures

**Figure 4:**
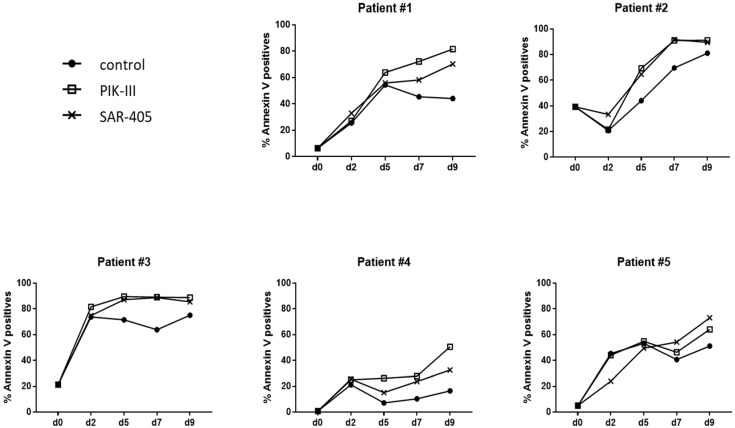
FLT3-ITD primary AML cells undergo apoptosis back to atmospheric O_2_ concentration. Primary AML blasts (2.10^5^) from 5 AML patients were incubated for 2 days at 1% O_2_ in co-culture on stromal cells and then placed at 20% O_2_ with vehicle, PIK-III or SAR-405, for 7 days_._ Every 2 days, an aliquot was analyzed by flow cytometry to measure apoptosis using Annexin-V APC. Results are shown individually for each AML patient.

To confirm these results on primary AML cells, we selected primary cells from five AML patients harboring the FLT3-ITD mutation (Table 1). Samples were chosen with a high percent of blast cells and an FLT3-ITD ratio above 42%. AML patients’ characteristics are presented in Table 1. Primary blasts were incubated for 2 days at 1% O_2_ in co-culture with stromal cells MS5 and then at 20% O_2_ on MS5 cells with or without PIK-III or SAR-405 (Figure 4).

Detection of apoptosis by annexin V labeling every 2 days demonstrated that at 20% O_2_, AML cells of all AML patients underwent an increase in apoptosis, while autophagy was inhibited (Figure 4), even though this increase was not significant when the responses of the five AML patients were pooled (Supplementary Figure S4A,B).

### 3.5. In Vivo, Combined Inhibition of Autophagy by PIK-III and SAR-405 and Mobilization Decrease Relapse Rate in Xenograft Model of FLT3-ITD AML Cells

**Figure 5:**
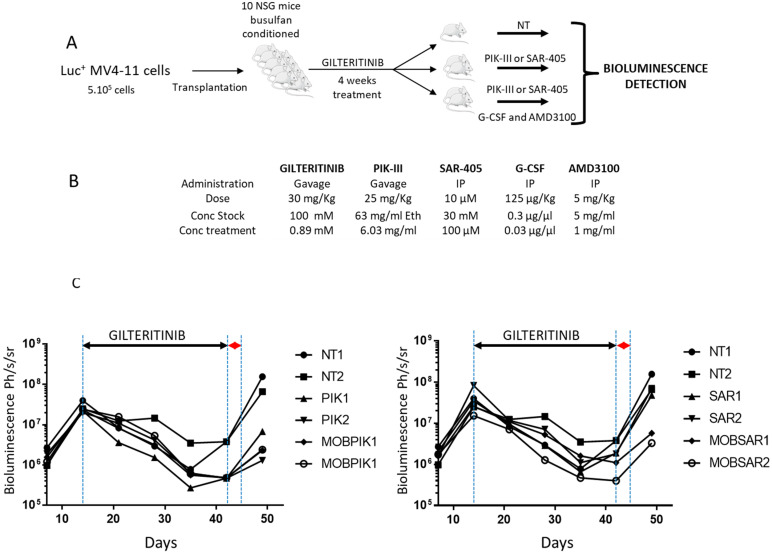
Inhibition of autophagy by Vps34 inhibitors and mobilization decrease relapse rate in vivo. (**A**) NSG mice were xenografted by IV injection of MV4-11 cells (5 × 10^5^). After two weeks, mice were treated by daily oral gavage of gilteritinib (30 mg/kg). After 4 weeks of treatment, mice were randomized and treated for two days as follows (**B**) NT (not treated), SAR (SAR-405 10 µM IP), MSAR (SAR-405 10 µM, G-CSF 125 µg/kg, AMD3100 5 mg/mL IP), PIK (PIK 25 mg/kg gavage) and MPIK (PIK 25 mg/kg gavage, G-CSF 125 µg/kg IP, AMD3100 5 mg/mL IP). (**C**) Bioluminescence was performed every 7 days (d_7_,d_14_,d_21_, d_28_, d_35_, d_42_ and d_49_).

To confirm the role of autophagy in the repopulating capacity of FLT3-ITD cells, we used MV4-11 engineered to express luciferase. We engrafted immuno-deficient mice by the IV injection of five 10^5^ cells. By bioluminescence imaging, leukemogenesis was quantified every 7 days according to the procedure shown in Figure 5A. Mice were treated daily from day 14 by gilteritinib (ASP2215, 30 mg/kg, a dual FLT3/AXL inhibitor) for 4 weeks (Figure 5B). Reduced leukemic burden was followed by bioluminescence imaging. At day 42, mice were randomized upon bioluminescence imaging and gilteritinib treatment was discontinued. A minimal residual bioluminescence signal was detected for all mice after 4 weeks of gilteritinib treatment, confirming the efficacy of gilteritinib (Figure 5C).

After randomization and discontinuation of the gilteritinib treatment, mice were either left untreated or were treated by PIK-III or SAR-405, with or without mobilization by G-CSF and AMD3100 for 2 days. After 7 days (d_49_), untreated mice had relapsed rapidly while PIK-III- and SAR-405-treated mice had relapsed slowly. Interestingly, the combination of mobilizing agents and Vps34 inhibitors induced a strong decrease in the relapse rate. While two mice treated by SAR-405 died, the experiment continued until d_53_ to confirm the reduction in relapse rate by the mobilization and inhibition of Vps34 (Supplementary Figure S5). The relapsing mice showed a very high BLI signal, while mice treated by a combination of mobilizing agent and Vps34 inhibitor showed a very weak signal.

## 4. Discussion

Targeting tyrosine kinase-driving leukemogenesis has become a gold standard since the development of targeted therapies using TKI, such as in chronic myeloid leukemia 20 years ago. In FLT3-ITD-driven AML, the kinase has been targeted by several TKI, such as midostaurin, quizartinib and, recently, gilteritinib [37,38,39,40]. Clinical assays demonstrate patient responses, which can reach complete remission with minimal residual disease. However, relapse still occurs even if delayed and is often associated with additional molecular anomalies. The dual inhibitor gilteritinib targets both FLT3 and AXL [41]. In our hands, it induces a strong and deeper response both in vitro and in vivo and is well tolerated in xenografted mice after 4 weeks of treatment. Recently, results from Joshi et al. show different early and late resistance mechanisms to gilteritinib [42]. Identification of aurora kinase involvement will allow us to reach CR in resistant patients and then to target FLT3-ITD LIC by the combination of Vps34 inhibitors and mobilizing agents.

To test the hypothesis that the leukemic cells remaining after TKI treatment are not fully erased, we explored the repopulating capacity of FLT3-ITD cells mimicking cells leaving the hematopoietic niche. Indeed, in vitro using AML cell lines, we demonstrate an important dependence on autophagy for FLT3-ITD cells to be able to repopulate. All quantitative results in vitro significantly demonstrated that autophagy is required for FLT3-ITD cells to be able to repopulate. Because each AML cell line used is p53 wild type, the autophagy inhibition upon 7 days in hypoxia, which also induce p53, is increasing p53 induced-apoptosis [43]. Indeed, cytoprotective autophagy is often associated with chemoresistance, as shown, for example, in colon cancer cells [44]. A similar behavior was also observed for primary AML blast cells despite non-significant results, although the small number of patients may explain this finding. A similar antileukemic activity of the Vps34 inhibitor has been reported for a close PIK-III inhibitor (INI-1) through STAT5 inhibition while sparing normal progenitor cells [45].

More importantly, in vivo engrafted mice with FLT3-ITD cells responded well to the FLT3/AXL inhibitor gilteritinib and achieved minimal residual disease with no longer any significant bioluminescent signal after 4 weeks of treatment. Following 2 days of treatment using Vps34 inhibitors, a decrease in relapse rate was observed for both PIK-III and SAR-405. A stronger decrease was observed when these inhibitors were combined with the mobilizing agents G-CSF and AMD3100. This confirms what was already reported about the mobilization of hematopoietic cells pushing cells out of their protective niche and helping to target leukemic cells by the autophagy inhibition, which blocks their capacity to repopulate [22,23,46]. Indeed, the Vps34 inhibitor PIK-III decreases the relapse rate with or without mobilizing agents. This can be explained by a different level of autophagy inhibition, off-target effects or the different way of administration. PIK-III has also been reported to reduce the number of primary CML cells, while chloroquine does not [47]. In contrast, chloroquine or Bafilomycin A1 have been implicated in the targeting of AML leukemic stem cells by disrupting mitochondrial homeostasis [48].

Because AML FLT3-ITD cells are known to require autophagy, the inhibition of FLT3-ITD cells by FLT3 TKI decrease the disease level strongly enough to reach complete remission, then allowing autophagy to be blocked. Comparing MV4-11 (FLT3-ITD) with MOLM-14, which is heterozygous for FLT3 and Oci-AML3 and independent of FLT3, confirmed that FLT3-ITD cells fully require autophagy when leaving the hematopoietic niche. Surprisingly, both inhibitors of Vps34 also decrease the repopulating capacity of MOLM-14 and Oci-AML3 in vitro, suggesting that while they are more efficient on FLT3-ITD cells, they could also be administered to keep the disease at a low level.

The combination with mobilizing agents would also promote the growth of healthy hematopoietic stem cells [49]. In addition, gilteritinib treatment has been reported to spare normal hematopoiesis [50]. This would help in a larger proportion of AML patients in remission. By combining gilteritinib with an autophagy inhibitor, only AML FLT3-ITD cells would be targeted and eliminated. Then, when complete remission is reached, a combination of Vps34 inhibition and mobilization should eliminate the remaining leukemic cells, although the cost–benefit ratio of this strategy should be carefully evaluated. Theoretically, this combination would greatly improve the relapse rate when mobilizing agents are associated with Vps34 inhibitors.

## 5. Conclusions

In summary, autophagy inhibition prevents the repopulating capacity of FLT3-ITD cells in vitro and ex vivo. A strong decrease is observed in vivo when autophagy inhibition is combined to mobilization. A study should be undertaken to identify the optimal mode of administration and which therapeutic window will be more efficient.

It can be worthwhile to check the different associations or combinations and, in particular, the opportune moment to administer mobilizing agents and/or autophagy inhibitors in vivo. This will require a sensitive method to measure the minimal residual disease to ensure perfect timing and dosing.

## Figures and Tables

**Table 1:** Biological characteristics of AML patients.

Patients N°	Sex	Age	Ratio	% Blasts	Mutation	Phenotype
1	w	57	45	70	NPM1	AML4
2	w	*74*	42	83	NPM1	AML4
3	w	37	170	79	NPM1	AML1
4	w	71	72	98	NPM1	AML1
5	m	49	48	89	NPM1	AML5

AML patient samples from the CRB Cancer Centre in Bordeaux were anonymously numbered and information concerning sexes, age FLT3-ITD ratio, number of blast cells, mutation panel and FAB phenotype were collected. Biological characteristics of AML patients: w = woman, m = man, ratio: FLT3-ITD/FLT3WT.

## Data Availability

Data available on request due to restrictions eg privacy or ethical The data presented in this study are available on request from the corresponding author. The data are not publicly available due to INPI letter submission DSO2017006047.

## Supplementary Materials

The following are available online at https://www.mdpi.com/article/10.3390/cancers14020453/s1, Figure S1: Vps34 inhibition block autophagy in FLT3-ITD AML cell lines. AML cell lines in which mCherry-GFP-LC3 was expressed were sorted using the GFP and mCherry expression and were analyzed by flow cytometry as a homogenous cell population with a purity above 98%. (A–C) Cells (10^5^ cells) were incubated with increasing concentrations of PIK-III or SAR-405 for 24 h in 500 μL of a-MEM. Both Vps34 inhibitors increased GFP and mCherry fluorescence in a dose-dependent manner. D: GFP fluorescence was observed on a PAULA microscope for MV4-11 cells treated with increasing concentrations of PIK-III. E: AML cell lines were treated with PIK-III 3 µM or SAR-405 5 µM for 24 h until day 7, as described in Figure 1. The cells were lysed and the inhibition of autophagy by PIK-III and SAR-405 was analyzed by Western blotting detecting p62, Beclin and LC3. Antibody against actin was used as a loading control (A-2066, Sigma, Saint Quentin Fallavier, France). The result is representative of two experiments. Antibodies against p62 (H290), Beclin (612113) and LC3 (PA1-16931) were from Santa Cruz (Bergheimer, Germany), BD (Becton Dickinson, France) and Sigma (St Quentin Fallavier, France), respectively; Figure S2: (A) At indicated times (d_0_, d_7_ and d_14_), aliquots were analyzed for cell count by trypan blue exclusion assay. Results are from at least five experiments. Significance was quantitated using Wilcoxon test and is shown by an asterisk (* *p* < 0.05, ** *p* < 0.01, *** *p* < 0.001). (B) AML cell lines were lysed at day 14 upon the addition at day 7 of PIK-III 3 µM or SAR-405 5 µM, as described in Figure 1. Induced apoptosis was detected by Western blotting through the detection of PARP and caspase 3 cleavage. Actin was used as a loading control (A-2066, Sigma, Saint Quentin Fallavier, France). The result is representative of two experiments. Antibodies against PARP (11-835-238-001) and caspase 3 (8G10) were from Sigma (St Quentin Fallavier, France) and Sigma (Danvers, MA, USA), respectively; Figure S3: Inhibition of autophagy reduces repopulating capacity and CFC of FLT3-ITD AML cells. AML cells were incubated for 7 days at 1% O_2_ and then treated or not for 7 more days at 20% O_2_. After cell counting at day 14, 10^3^ cells were seeded in methylcellulose and incubated at 37 °C at 20% O_2_. After 14 days in methyl cellulose, colony number was quantified. (A) Observation by microscopy of colonies in methylcellulose. (B) Examples of CFC assays; Figure S4: Repopulating capacity of FLT3-ITD primary AML cells decreases by autophagy inhibition correlated with an increase in apoptosis. Primary AML blasts (2 × 10^5^) were incubated for 2 days at 1% O_2_ in co-culture on stromal cells and then treated or not by PIK-III or SAR-405 for 7 more days at 20% O_2._ Every 2 days, an aliquot was analyzed by flow cytometry to measure apoptosis using Annexin-V APC. (A) The results shown are the mean of five experiments. (B) Results are shown for AML patients at days 0, 2, 7 and 9; Figure S5: Inhibition of autophagy by Vps34 inhibitors and mobilization decrease relapse rate in vivo. NSG mice were xenografted by IV injection of MV4-11 cells (5 × 10^5^). After two weeks, mice were treated by daily oral gavage of Gilteritinib (30 mg/kg). After 4 weeks of treatment, mice were randomized and treated for two days as follows: NT (not treated), SAR (SAR-405 10 µM IP), MSAR (SAR-405 10 µM, G-CSF 125 µg/kg, AMD3100 5 mg/mL IP), PIK (PIK 25 mg/kg gavage) and MPIK (PIK 25 mg/kg gavage, G-CSF 125 µg/kg IP, AMD3100 5 mg/mL IP). Bioluminescence was performed every 7 days (d_7_, d_14_, d_21_, d_28_, d_35_, d_42_, d_49_ and d_53_).
