# Supplementary material for: Autophagy Targeting and Hematological Mobilization in FLT3-ITD Acute Myeloid Leukemia Decrease Repopulating Capacity and Relapse by Inducing Apoptosis of Committed Leukemic Cells
Source: Cancers (Basel). 2022 Jan 17;14(2):453. doi: 10.3390/cancers14020453 (PMC8796021; doi:10.3390/cancers14020453)
Supplement: Supplementary file 1 [file cancers-14-00453-s001.zip › supplementary cancers-1511083.pdf]

Figure S1A

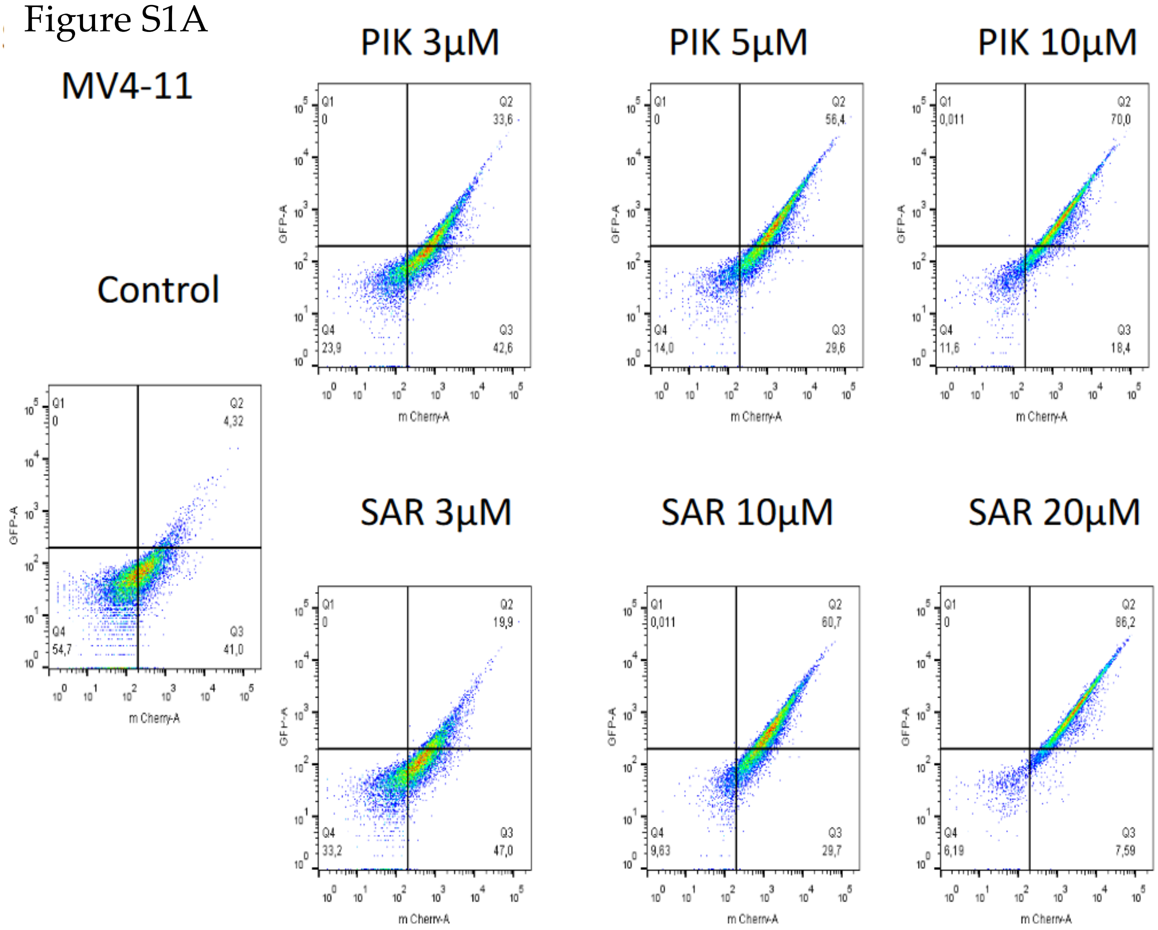

Figure S1B

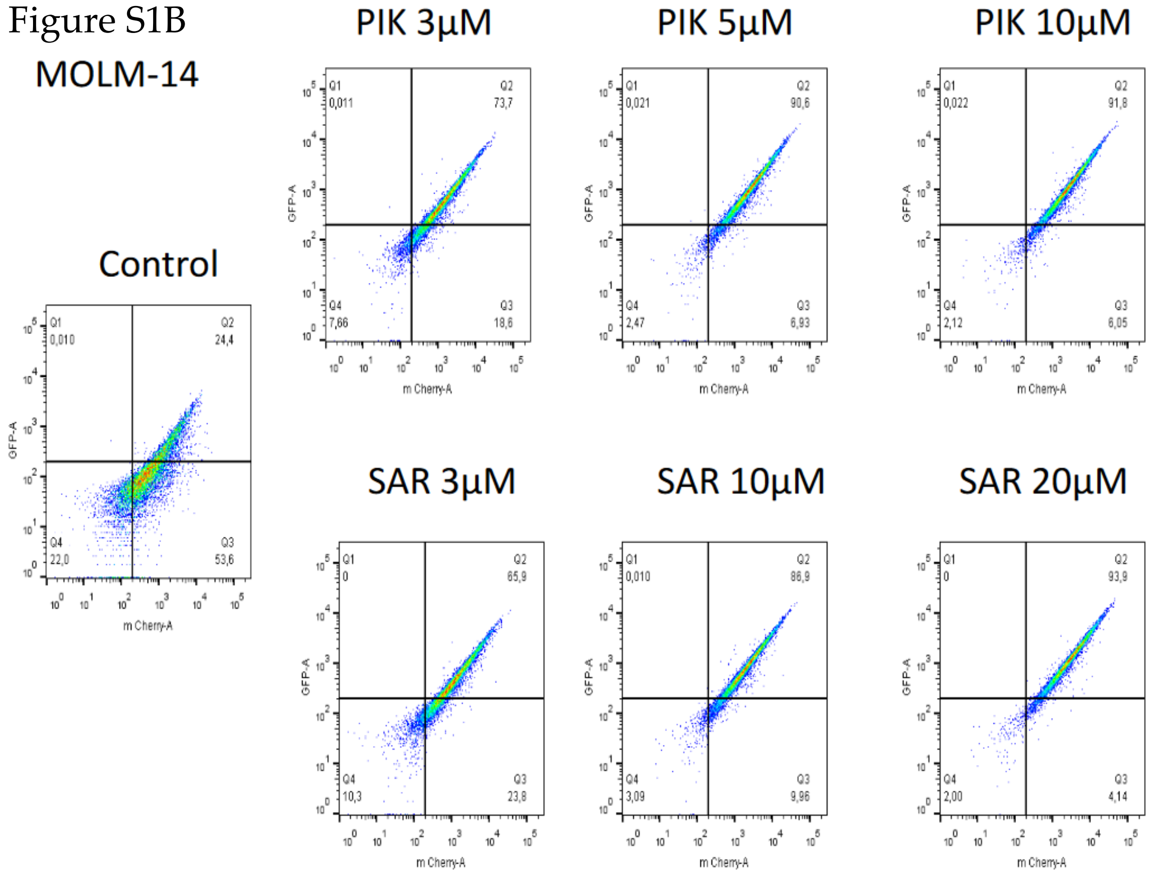

Figure S1C

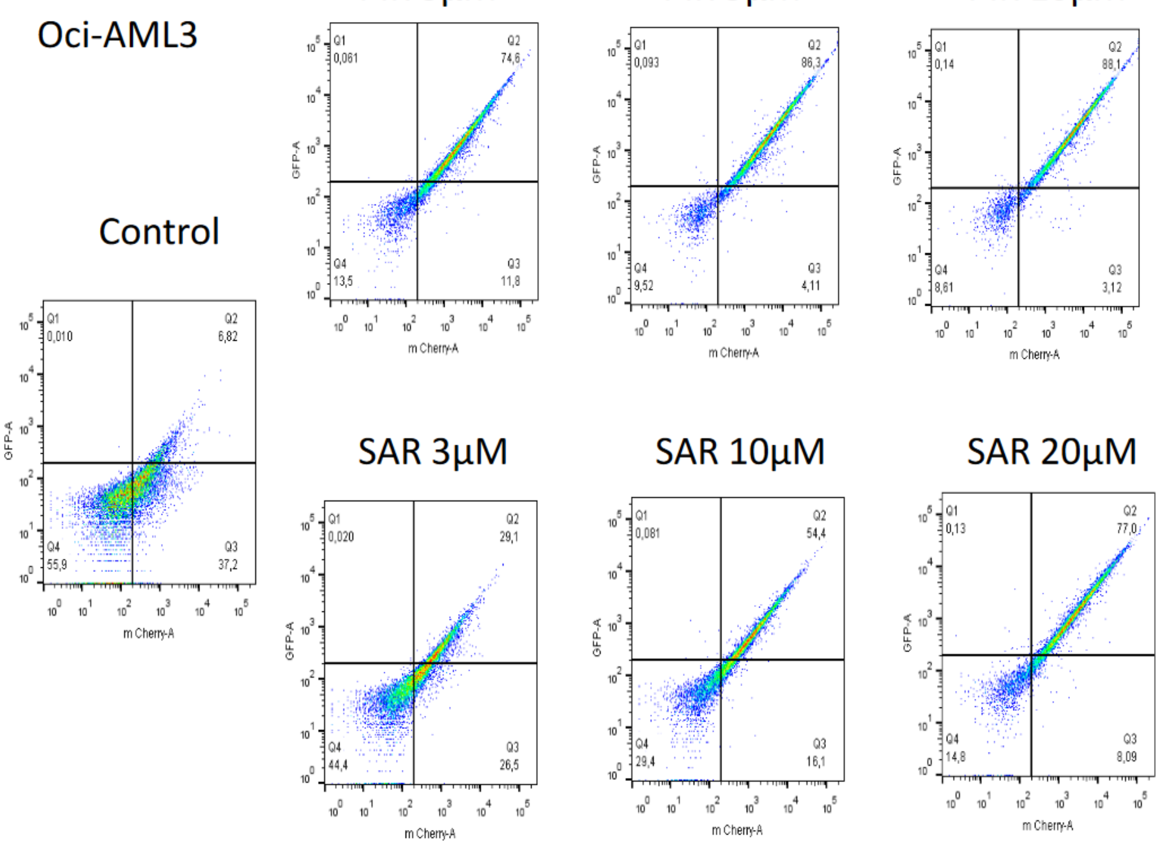

Figure S1D

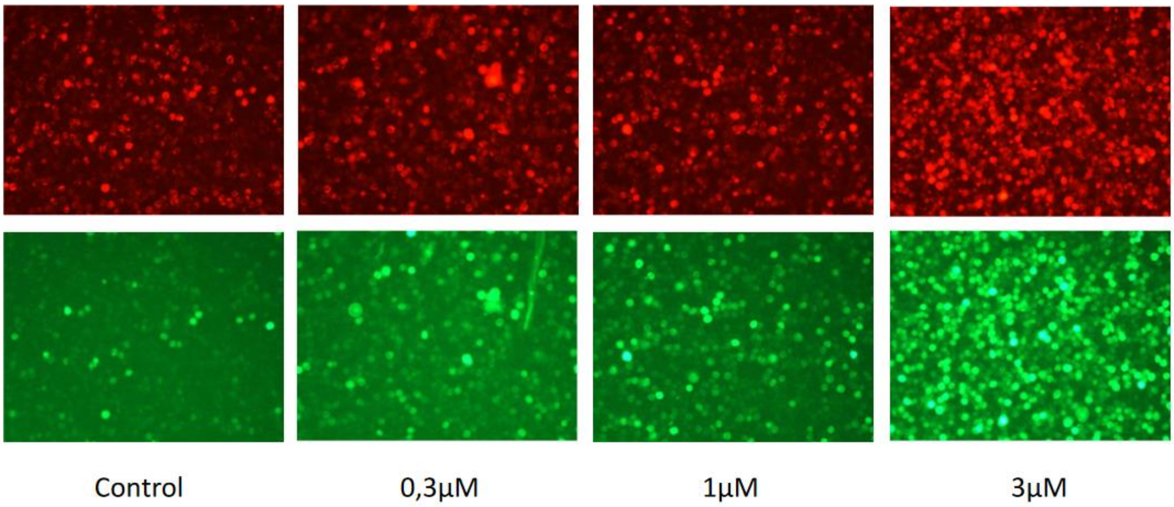

MV4-11 mCherry-GFP → Inhibitor PIK – III

Figure S1E

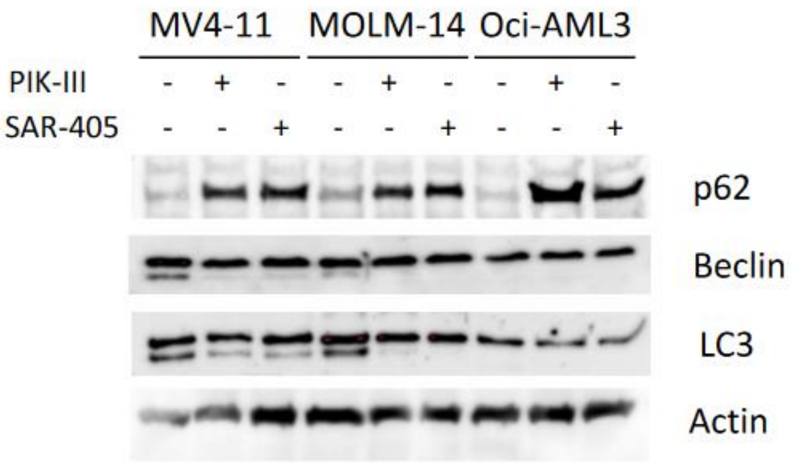

Figure S2A

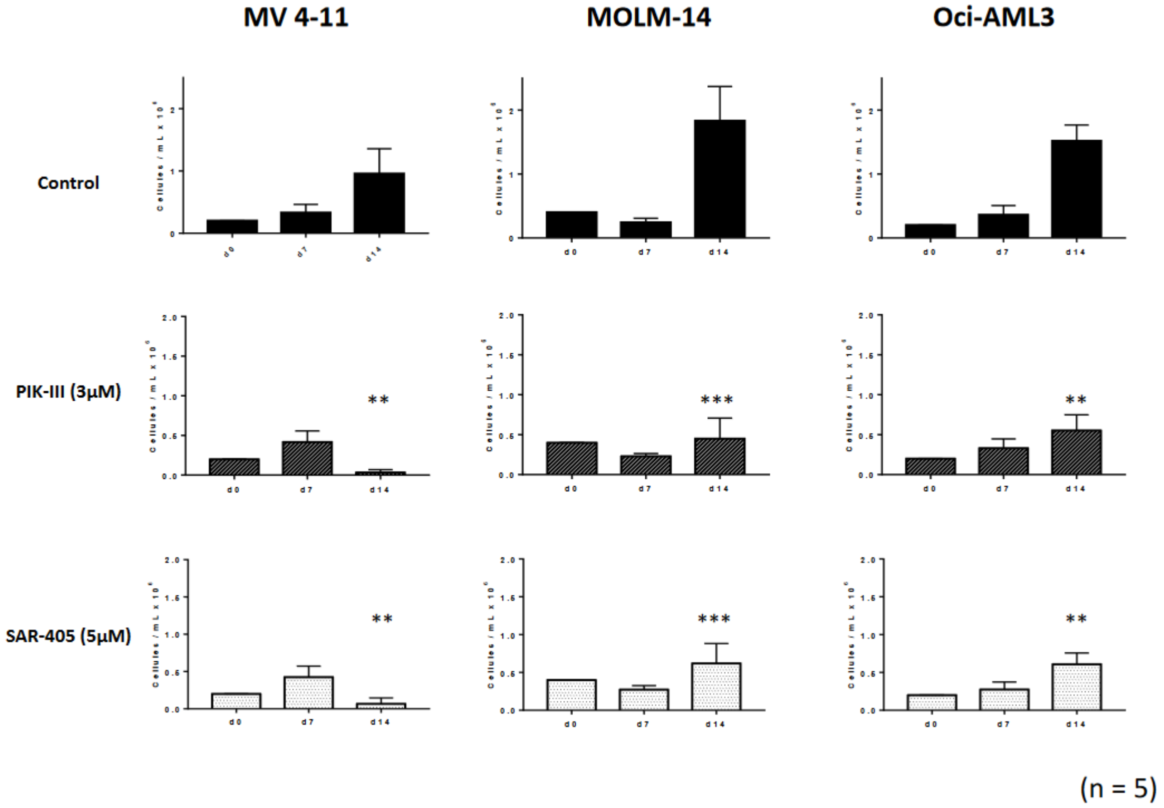

Figure S2B

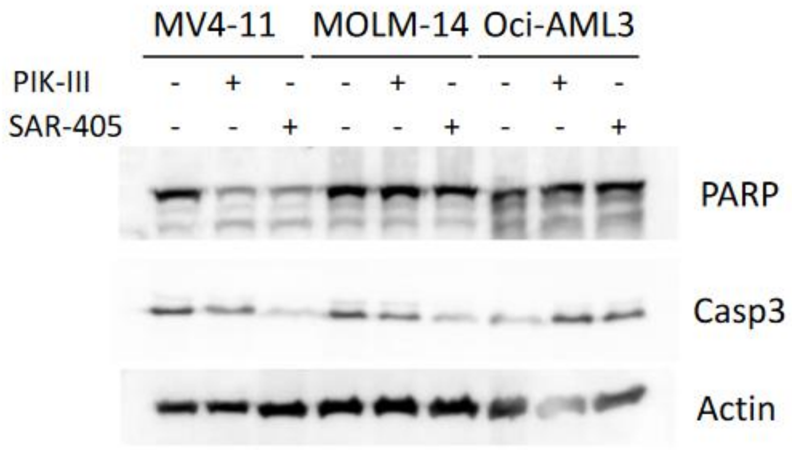

Figure S3A

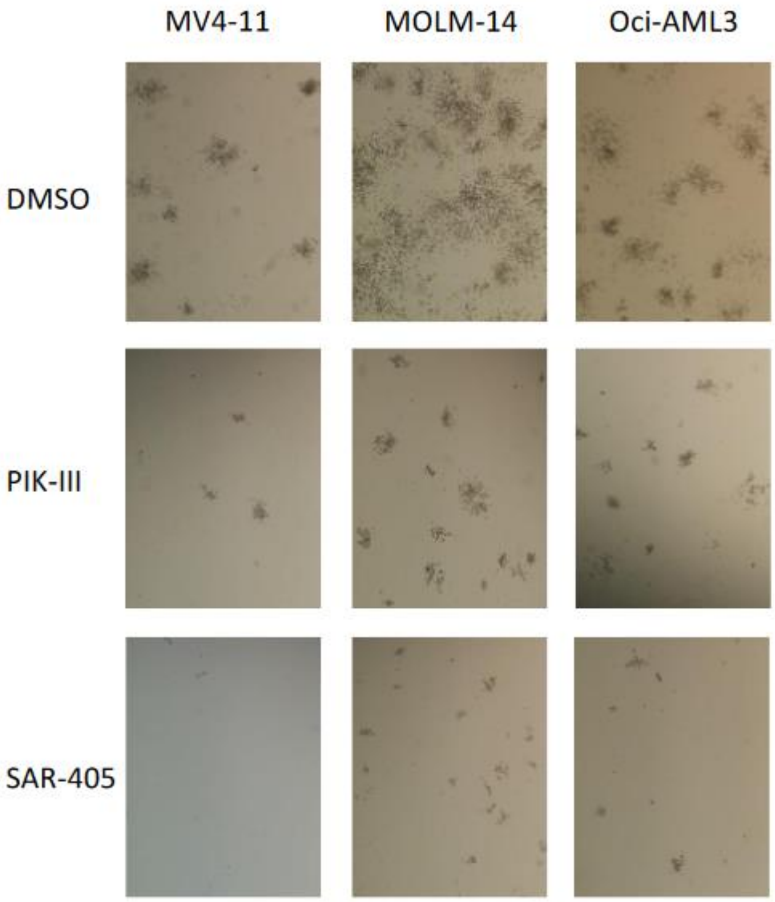

Figure S3B

Two differents experiments illustrating CFC assay

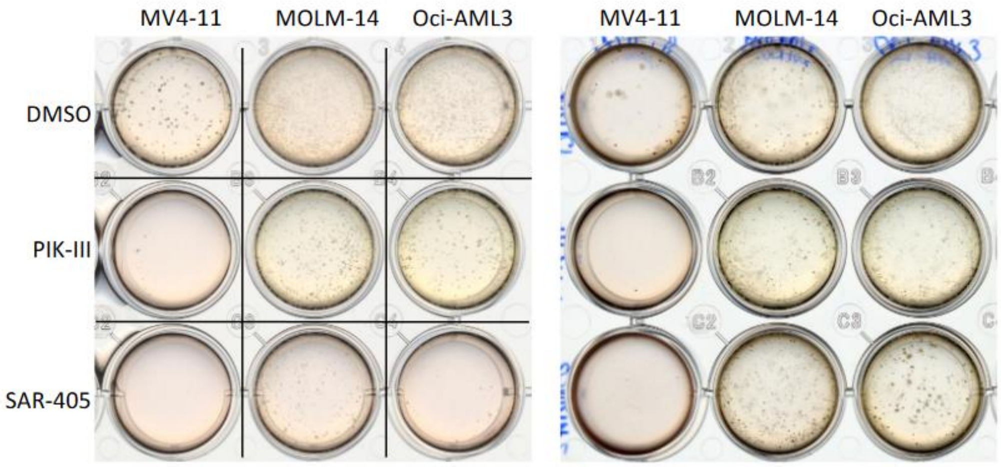

Figure S4A

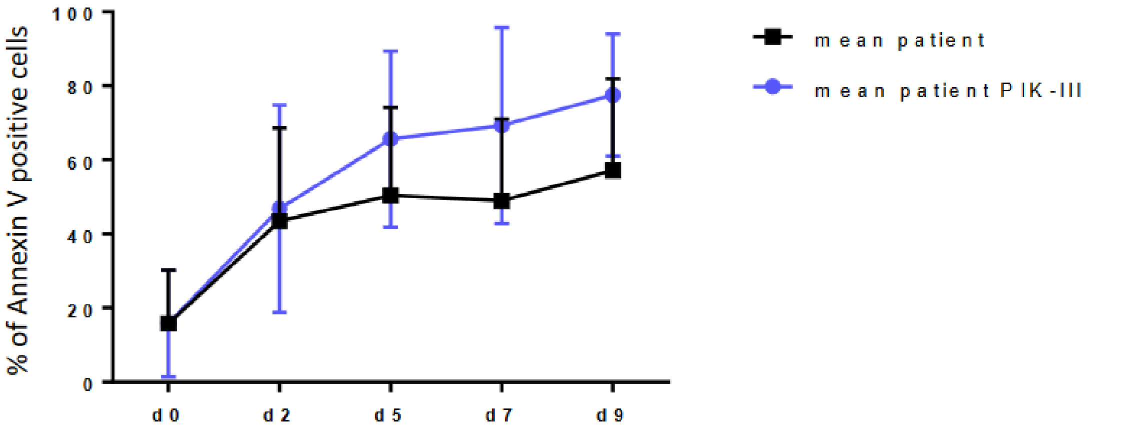

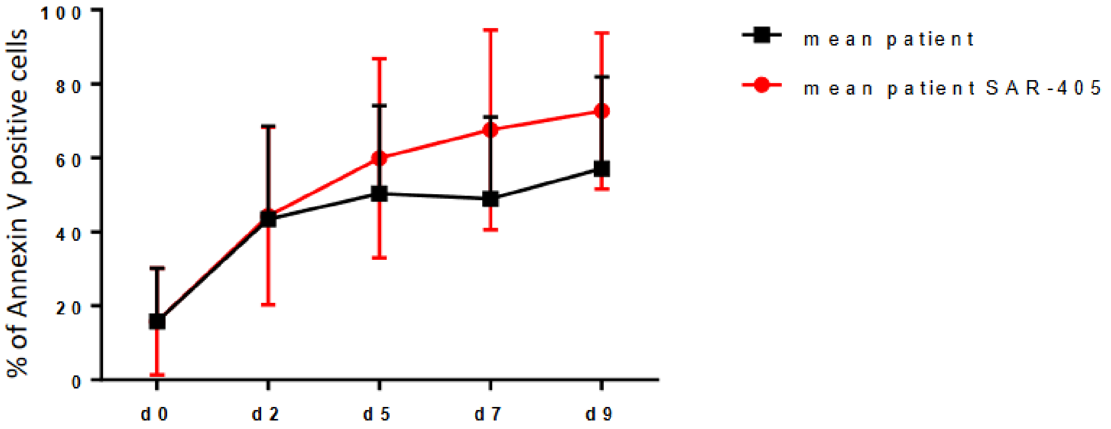

Figure S4B

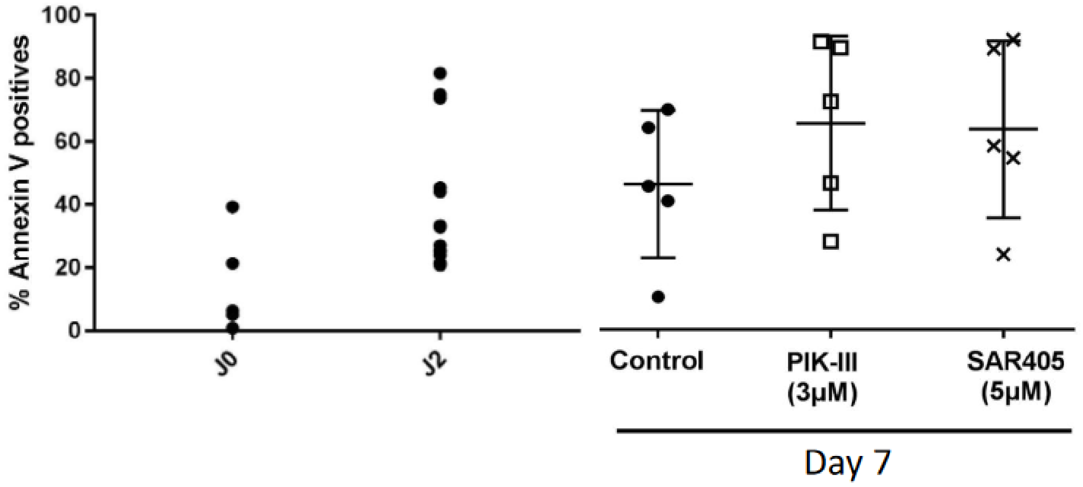
% Annexin V positives
100
80
60
40
20
0
J0
J2
Control
PIK-III (3µM)
SAR405 (5µM)
Day 7

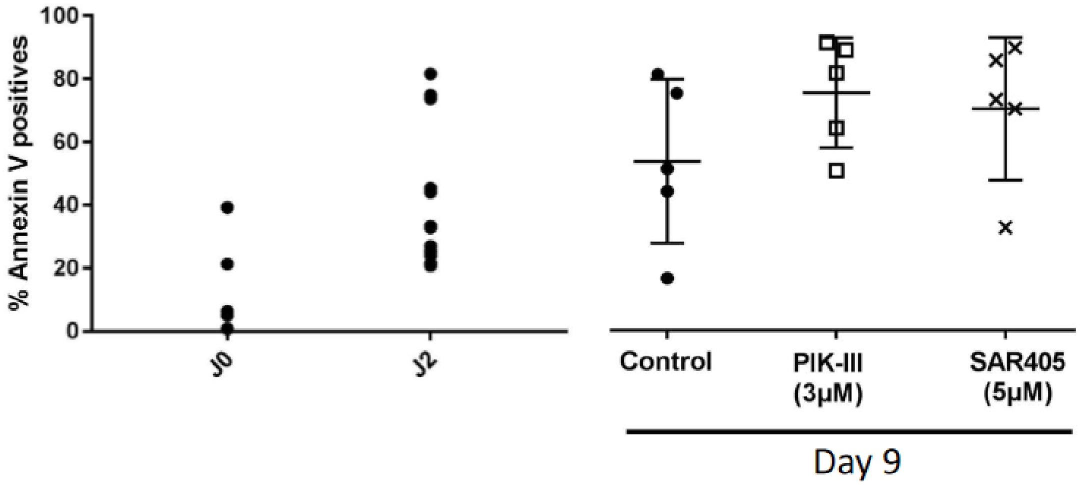
% Annexin V positives
100
80
60
40
20
0
J0
J2
Control
PIK-III (3µM)
SAR405 (5µM)
Day 9

Figure S5

Full time experiment day 53

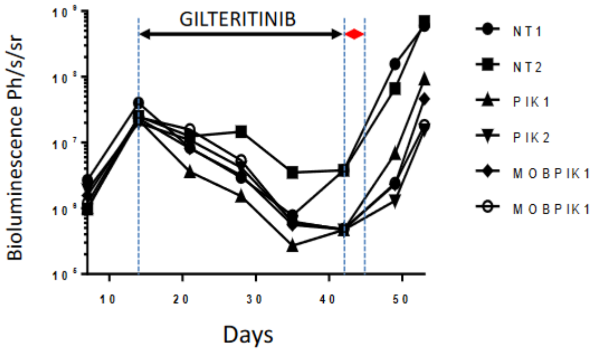

PIK III +/- MOB

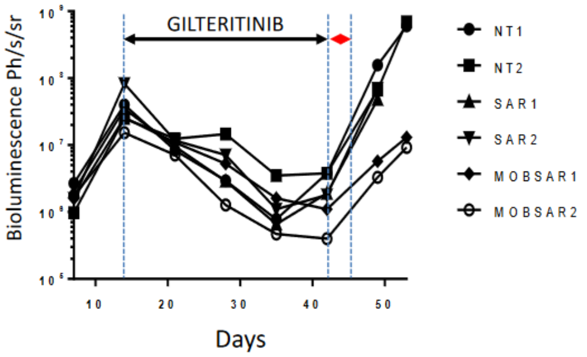

SAR-405 +/- MOB

Western-blots

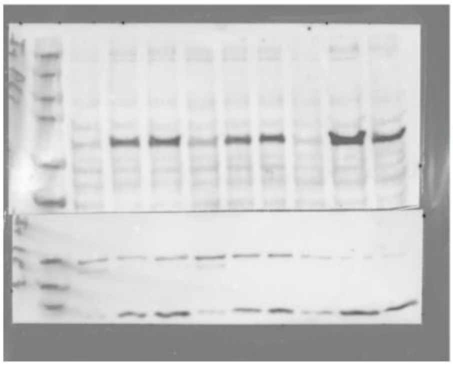

p62

LC3

Hsp 60

Actin

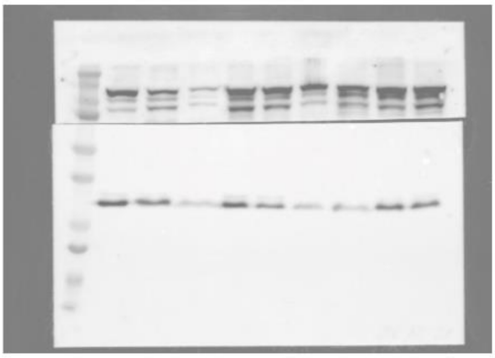

PARP

Casp

Hsp 60

Actin

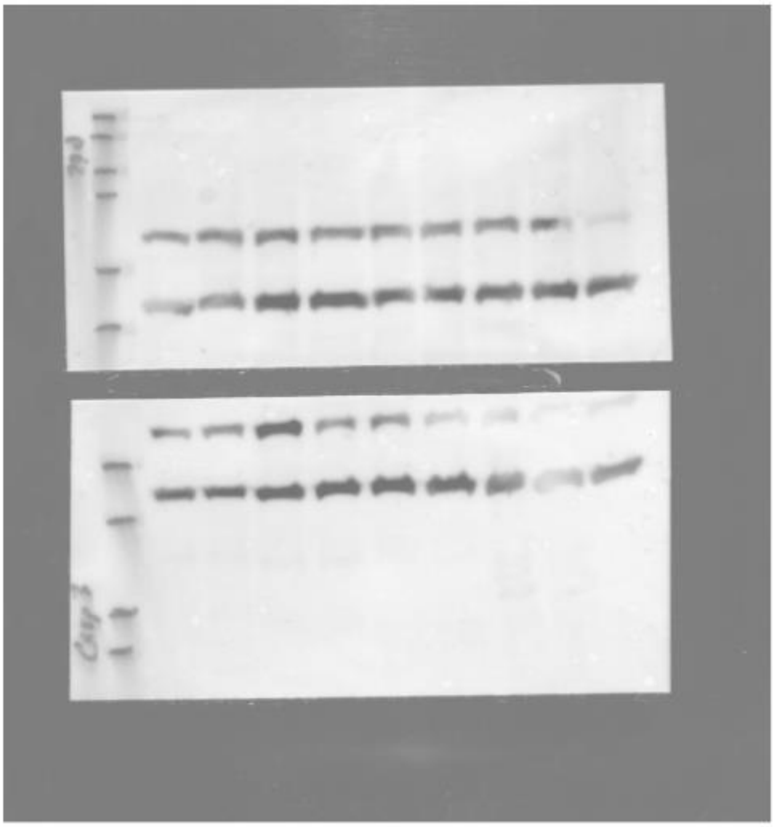

Hsp 60

Actin

Hsp 60

Actin
